# An Assessment of the Micro-Tensile Bond Strength of Composites for Indirect Restoration to Enamel and Dentin

**DOI:** 10.3390/jfb16040138

**Published:** 2025-04-12

**Authors:** Viktoria Petrova, Janet Kirilova, Sevda Yantcheva

**Affiliations:** Department of Conservative Dentistry, Faculty of Dental Medicine, Medical University, 1000 Sofia, Bulgaria; nikolova@fdm.mu-sofia.bg (J.K.); s.yancheva@fdm.mu-sofia.bg (S.Y.)

**Keywords:** micro-tensile bond strength, composite inlay, CAD/CAM, restorative dentistry

## Abstract

This study aimed to evaluate the micro-tensile bond strength (µTBS) of two types of composites for indirect restoration, luted to enamel and dentin with self-adhesive cement. Moreover, it aimed to evaluate the impact of thermocycling on bond strength. Sixteen flat enamel and dentin surfaces of human molars were cemented to equal flat specimens of the laboratory composite Signum ceramis and the CAD/CAM block Cerasmart. Half of the specimens of the group underwent thermocycling. After that, the samples were cut into 80 beams for µTBS analysis. The data were analyzed using Levene’s test and the independent sample t-test. The micro-tensile bond strength tests revealed that thermocycling significantly reduced the adhesive bond. Dentin bonds better to conventional laboratory composites. Enamel bonds are better than composite blocks for milling.

## 1. Introduction

The use of dental amalgams is limited, tending toward complete removal when used as a restorative material. Direct and indirect composites [1,2] are used for restoring vital posterior teeth with extensive destruction, corresponding to a minimally invasive work technique. Despite the progress in the development of direct composite materials, they still have several disadvantages that make them unsuitable for obturation with significant loss of hard dental tissues. Polymerization shrinkage is a major problem, leading to the formation of a marginal gap, microleakage, and secondary carious lesions [3,4]. The linear polymerization shrinkage of composite materials containing Bis-GMA is between 0.3 and 1.5% [5], and the volumetric shrinkage is 1.5–3.5% [6].

Composite materials for indirect restorations are divided into two groups: laboratory (used by dental technicians for layer-by-layer restorations) and computer-aided design/computer-aided manufacturing (CAD/CAM) blocks, which are milled by machines. The latest composite materials for indirect restorations, such as Cerasmart (*GC Europe, Leuven, Belgium*), can be called hybrid ceramics, but their composition is similar to direct composites [7]. Indirect restoration methods improve composite materials’ physicochemical properties, reducing polymerization shrinkage and marginal gaps. These types of materials are prepolymerized [8], and the changes regarding shrinkage occur only in the thin layer of the cement. Laboratory composites comprise two generations. The first one is presented by Touati and Morman, including Brilliant (*Coltene*) and Concept (*Ivoclar*) [9]. The unsatisfactory results of this type of composite result from the insufficient bonding between the organic matrix and the inorganic fillers [10]. This leads to increases in the frequency of fractures, the formation of a marginal gap, microleakage, and poor adhesion of the obturation in the distal area.

The second generation is more widely used in dental practice, such as Signum ceramis (Heraeus Kulzer), Artglass (Heraeus Kulzer), and Belleglass HP (Kerr). These are hybrid materials combining the qualities of ceramic and composite materials [10]. The inorganic component in their composition is approximately twice as large as the organic component, which improves their mechanical properties and wear resistance.

With the improvement of dental medicine technologies, CAD/CAM systems emerged in the 1980s [11,12]. The computer-generated design for inlays, overlays, and onlays is fed into a CAD/CAM machine. The machine mills the final restoration from composite blocks. This improves the accuracy of the obtained restoration to the cavity shape, adjacent teeth, and antagonists [13]. The first composite blocks for CAD/CAM machines were introduced in 2000. Their content is similar to direct composite materials, but their light polymerization is performed in the factory [14,15]. This significantly improves their mechanical properties and reduces their shrinkage [16]. The retention of indirect restorations is mainly achieved via adhesion [17]. The strength of the adhesive bond is crucial for the longevity of indirect restorations. The adhesion of conventional CAD/CAM restorations is directly related to their cementation to the hard dental tissues [18,19,20,21]. In recent years, self-adhesive dual-cure cement is most often used to bond aesthetic indirect restoration [18,20,22]. There is a lack of data in the literature regarding the investigation of the strength of the adhesive bond of new composite materials (conventional and milling blocks) after thermocycling. Data regarding the bond with different dental structures, such as enamel and dentin, are also insufficient. This study aimed to evaluate the tensile bond strength of the adhesive bond between enamel or dentin and two composite materials reinforced with ceramic particles, including laboratory and milling blocks, with and without thermocycling. The null hypotheses tested were as follows:

The thermocycling does not affect the bond strength.

The micro-tensile bond strength of the CAD/CAM material Cerasmart is similar to the laboratory composite Signum ceramis.

## 2. Materials and Methods

The Medical University–Sofia Research Ethics Committee approved this project (approval number: 53). Thirty-two human molars extracted within 3 months were used for this in vitro study. The remaining hard and soft dental tissue was cleaned with periodontal curettes and kept in distilled water with a 0.1% thymol solution. The micro-tensile bond strength (µTBS) was evaluated on 80 specimens made of enamel/dentin and two types of composite materials for indirect restorations: CERASMART milling blocks (GC Europe, Leuven, Belgium) and the conventional laboratory composite Signum ceramis (Heraeus Kulzer, Hanau, Germany) (Table 1).

### 2.1. Fabrication of Composite Samples

One of the composites, CERASMART (GC Europe, Leuven, Belgium), represented the CAD/CAM blocks. Sixteen cylindrical standard models with a height of 4 mm and a length of 8 mm were made from them. The 4 mm thick slabs were obtained from the tested rectangular-shaped blocks with a diamond saw (LEICA SP 1600). The specimens were measured with a digital caliper. The other material was the conventional laboratory composite Signum ceramis (Heraeus Kulzer, Hanau, Germany). The material was initially polymerized in a mold made from Cerasmart samples. The Cerasmart samples were placed in silicone to create a mold for the conventional laboratory composite, Signum ceramis. After the silicone mass hardened, the milling material was removed, and the traditional laboratory composite, Signum ceramis, was sequentially applied to the mold in two portions. Each portion of Signum ceramis was polymerized for 6 s. After removing the specimen from the mold, a final polymerization was performed for 90 s in a Hi-Light power 3D (Heraeus Kulzer, Hanau, Germany) oven. This technology made sixteen specimens from each group of composite material.

### 2.2. Preparation of Enamel Specimens

**Figure 1:**
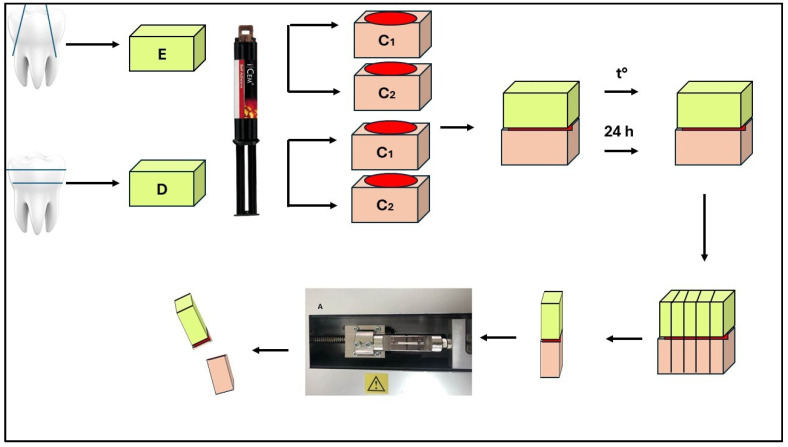
Scheme for preparing enamel and dentin specimens for micro-tensile testing. Legend: E—enamel; D—dentin; C_1_—composite 1 (Signum ceramis); C_2_—composite 2 (Cerasmart); and t°—thermocycling.

The enamel surfaces of 16 molars were separated buccally and lingually using a diamond bur. The lingual/buccal enamel of the molars was flatted using a high-speed, medium-grit (100 µm) diamond bur (Edenta AG, Au, Switzerland). Then, they were divided into four equal groups (×4 specimens in each). Composite blocks with a height of 4 mm from the laboratory composite Signum ceramis were cemented to groups A and B, and CAD/CAM blocks (CERASMART) with a height of 4 mm using the self-adhesive cement iCEM (Heraeus Kulzer, Hanau, Germany) were cemented to groups C and D. The enamel samples were dry at the time of bonding. A light-curing unit was positioned 1 mm away from the specimens, and all were initially polymerized on all proximal sides and then from the top for 20 sec. The cut enamel surface was not treated (according to the manufacturer’s instructions). The composite materials were sandblasted with 50 µm of Al_2_O_3_ at 3 atm and a 10 mm distance for 20 s, followed by silanization. The test bodies from groups A and C were subjected to standardized thermal loading (for 1000 cycles) using a thermocycler (SD Mechatronik, Munich, Germany) at bath temperatures of 5 and 55 °C. They stayed in the hot and cold baths for 30 s, and the holding time before immersion in one of them was 15 s. After the thermocycling stage, each specimen was embedded in an epoxy block. This block was cut into three mutually perpendicular planes until miniature test beam-shaped specimens for physicomechanical testing were obtained using a Leica SP 1600 microtome under continuous water cooling. The dimensions of the square cross-section were 1 × 1 mm, and the height of the beams was 8 mm. The dimensions were checked with a digital caliper with a ± 0.1mm accuracy. Sticks from each tooth’s center area were used, while the sticks from the periphery were not. The beams from groups B and D were also embedded in resin but were not subjected to thermocycling aging. They were directly cut into specimens. For µTBS evaluation (Figure 1), the beams were appropriately positioned in an LMT 100 stand (LAM. Technologies, Firenze FI, Italy) and subjected to static tensile loading at a 0.5 mm/min speed. The registered maximum value of the force (N) resisting the test body, divided by its initial cross-sectional area (S), was the tensile strength, with a dimension of MPa. The conditions during the tensile test were a temperature of 23 ± 2 °C and humidity of 50 ± 5%RH.

### 2.3. Preparation of Dentin Specimens

The occlusal third on 16 teeth was removed using a diamond bur. The aim was to expose the dentin. The lower third of the crown was also removed. The middle third of the dentin surface was examined using a magnification of ×9 for the absence of enamel and pulp tissue. The prepared dentin specimens were divided into four equal groups (×4 specimens each). Composite blocks with a height of 4 mm from the laboratory composite Signum ceramis were cemented to groups E and F. CERASMART CAD/CAM blocks were cemented to groups G and H using the self-adhesive cement iCem. The dentin samples were dry at the time of bonding. The dentin surface did not require pretreatment according to the manufacturer’s instructions for the cementing agent, and the composite materials were sandblasted (using 50 µm of Al_2_O_3_) at 3 atm for 20 s, followed by silanization. The dentin specimens in groups E and F were similarly cemented to 8 composite Signum ceramis samples, and those in groups G and H to 8 CERASMART standards using the self-adhesive cement iCem. The dentin specimens from groups E and G, as the enamel specimens, were subjected to thermocycling loading under the conditions described. All specimens (including those from groups F and H) were individually embedded in activated epoxy resin and left for 72 h after the thermocycling effect. A similar preparation of specimens for determining the adhesive strength followed. The tests were performed under the same conditions, and the data obtained were processed similarly to the enamel specimens (Figure 1).

### 2.4. Statistical Analysis

Descriptive statistical techniques, such as mean and standard deviation values, were used to assess the data. The µTBS data were analyzed using Levene’s test for equality of variances and the independent samples t-test. All statistical analyses were carried out using IBM SPSS Statistics 24 (IBM, NY, USA). Statistical significance was set at a *p*-value of < 0.05 for all tests.

## 3. Results

The mean and standard deviations of the µTBS values are shown in Table 2. The independent samples t-test results revealed that thermocycling significantly affected the µTBS (*p* < 0.05) in all the groups except the Signum ceramis bond to dentin. Thermocycling reduced the tensile bond strength. Regarding Signum ceramis, the µTBS to enamel was significantly higher in enamel without thermocycling (12.22 ± 2.12) than after thermocycling (8.53 ± 1.93). The difference was not significant for the dentin specimens (*p* > 0.05). For Cerasmart, a statistically significant difference was observed for the enamel and dentin specimens with or without thermocycling (*p* < 0.05). A comparison of the results between the enamel/dentin specimens with/without thermocycling, cemented to the two types of composite material, is presented in Table 2. Regarding the enamel samples, the tensile bond strength was higher for composite 2. The data show the opposite for the dentin specimens: the tensile strength was higher for composite 1 (Signum ceramis). These data were statistically processed. The distribution in the groups was expected, and their dispersion was equal. The statistical processing results show no statistically significant difference in micro-tensile strength between composite 1 (Signum ceramis) and 2 (Cerasmart) cemented to enamel, regardless of whether they underwent thermocycling. The observed difference was random (non-systematic). However, a statistically significant difference in micro-tensile strength in favor of Signum ceramis was proven for dentin, regardless of whether the specimens underwent thermocycling. This second conclusion can be asserted with 99% probability. Summarizing the findings, it can be said that thermocycling led to fatigue of the hard dental tissue/composite material bond and a decrease in micro-tensile strength. This difference was statistically evident in three out of four tested tooth groups. It did not occur only in the dentin–composite 1 (Signum ceramis) bond. Meanwhile, the lowest micro-tensile strength values were observed after thermocycling the specimens made after cementing dentin to the Cerasmart composite block. Composite material 1 (Signum ceramis) had a stronger adhesive bond to dentin and composite material 2 (Cerasmart) to enamel.

## 4. Discussion

This in vitro study aimed to evaluate the µTBS of Signum ceramis and Cerasmart luted to enamel or dentin using dual-cure self-adhesive cement (iCem). The adhesion of indirect restorations to hard dental tissues is crucial for their longevity. Self-adhesive cement is the most widely used [23]. Most of the research in the literature concerns the study of the bond between the resin cement and enamel/dentin. The role of these bonding agents is very complex as they must establish a connection with the hard dental tissues and the composite material. Several quantitative methods for determining the strength of the adhesive bond have been described in the literature: shear strength, micro-shear strength, tensile strength, and micro-tensile strength [18,24,25,26,27,28]. Macro-testing requires an adhesion area exceeding 3 mm^2^, while micro-tests require less than 3 mm^2^ [29]. According to Griffith [30], the strength of the measured adhesive bond decreases with the increasing area of the tested specimens. Different methods for evaluating the strength of the adhesive bond also affect the results. Not many studies in the literature have used other tests to assess adhesion to enamel and dentin [18,24]. The specimens in this study were prepared according to the standardized guidelines and methodologies described in the literature [18,31,32]. Our study evaluated the adhesive strength by measuring the µTBS in MPa. Measuring micro-tensile strength is a more complex process involving the preparation of many specimens from a smaller amount of material. It is performed in a smaller area, contributing to a more uniform stress distribution [33]. Fewer material defects are observed on the smaller area of the specimens [24]. This methodology makes adhesive-type fractures more common and cohesive-type fractures less common [33]. This makes it a more reliable method of testing the bonding effectiveness of different restorative materials to hard dental tissues [34]. The micro-tensile strength values are higher than the macro-tensile strength values [24]. The adhesive strength found in the literature varies between 9 and 45.3 MPa and depends on the testing method and the composition of the material [35]. In our study, specimens that fractured before testing were not considered, and values of 0 MPa were not included. Some studies excluded these specimens [24,36], while others included them [37]. Enamel specimens are more prone to fracture before testing than dentin specimens. Enamel has a higher hardness than dentin (270–350 KHN and 50–70 KHN), and it is more fragile [38], which leads to the debonding of the hard dental tissues from the specimen during its cutting. In our study, specimens made of Cerasmart material bonded better to enamel than those made of Signum ceramis material. According to the µTBS results, the adhesion to enamel and dentin was significantly influenced by the type of materials, and the second null hypothesis was rejected. Since the experimental setup was performed under the same conditions for both groups, it can be argued that the Cerasmart material bonds more strongly to the self-adhesive cement iCEM. Adhesion to the enamel surface is more easily achieved and more reliable. Enamel comprises 94-96% inorganic components, 1–4% water, and 4–5% organic components [24]. It has higher intermolecular forces and a higher surface energy. Three models can be used to alter the structure of enamel with phosphoric acid and achieve adhesion: by attacking the prisms’ core, the prisms’ periphery, or a combination of both [24]. Modern self-adhesive cement does not require etching the enamel surface as it contains acidic methacrylate groups [39]. In our study, we followed this work protocol as in most studies found in the literature [35,40]. However, some authors reported higher micro-tensile strength values after the pretreatment of the enamel surface with 37% phosphoric acid [39].

Adhesion to dentin is more complex as it is a porous, moist structure consisting of hydroxyapatite particles in a collagen matrix. The organic component reduces the bond strength. Dentin tubules are wider and more permeable near the pulp. The water content is lower on the surface and increases at greater depths. In addition to these physiological differences, the dentin structure undergoes changes with increasing age or due to the presence of a carious process. An increase in thickness and a decrease in permeability are observed. All these factors make the dentin structure more complex for achieving satisfactory adhesion [25,28]. The micro-tensile strength values for dentin should be lower compared with enamel. In our study, the dentin surface was not treated with etching acid before the cementation of the composite material. The micro-tensile strength values for dentin cemented to composite material 1 (Signum ceramis) were higher than those for enamel. Similar results were reported in a review study by Scherrer et al. [41] and Bracher et al. [24]. According to the authors, the results were due to the formation of microcracks while cutting the enamel specimens. De Munck et al. found that a self-adhesive cement bond was better for dentin than enamel [39]. They suggested performing selective etching only of the enamel surface [39] as dentin treatment yields worse results than enamel treatment [39]. Further research on the selective etching of the enamel surface would improve the cementation protocol for indirect composite restorations.

The dentin and Signum ceramis samples showed the highest micro-tensile bond strength values. The dentin surface bonded better than the enamel surface with the tested cement. This dependence was not observed in the Cerasmart material samples. The enamel bonds were better than those of dentin with the resin cement. Since the primary goal in cavity preparation is to preserve enamel in the peripheral connection, cementation of Cerasmart restorations would yield a more reliable result.

The cement used in this study was self-adhesive. It did not require preliminary enamel/dentin surface treatment, which facilitated the work protocol and reduced postoperative sensitivity [40]. The bond formed with the tooth structure (dentin/enamel) was micro-mechanical and chemical. It was achieved between the cement’s multifunctional acid monomers and the tooth structure’s hydroxyapatite crystals [40]. Most studies report that despite the good qualities of self-adhesive cements, they still have a weaker adhesive bond than conventional cements, which require etching and applying a bonding agent [37,42,43]. Other studies show that self-adhesive types of cement bond are better than those requiring total etching [33,44]. Some authors reported that acid methacrylate esters cannot fully penetrate the partially dissolved smear layer. As a result, voids are formed, and the bond strength between the cement and dentin is reduced [45]. Self-adhesive cements are used for cementation in case of difficulties with isolation of the operative field, high-strength ceramics, and compromised retention when restoring teeth with little remaining hard dental tissues, mainly in dentin [46]. This explains the higher values we obtained for the dentin samples and Signum ceramis.

The treatment of the inner surface of indirect restorations also affects the adhesive bond strength. The composite material samples we prepared were first subjected to sandblasting with Al_2_O_3_, which achieved a micro-mechanical bond, and then silane was applied to achieve a chemical bond. Sandblasting with Al_2_O_3_ is a surface treatment method that aims to improve mechanical retention by creating microroughness [19,20]. There are different approaches to this procedure regarding particle size, pressure, and duration. Papadopoulos et al. and Tekce et al. recommended the particle size should not be larger than 50 μm [20,47]. Tekce et al. reported that a larger size of Al_2_O_3_ leads to cracks on the material surface [47]. The pressure during sandblasting affects the adhesive bond. Kim et al. reported that excessive pressure leads to the formation of sharp edges due to the concentration of tension in certain areas [48]. The authors advised using pressure between 1 and 2.5 bar [48]. Tekce et al. also stated that sandblasting should not exceed 30 sec due to the risk of cracking [47]. In this study, sandblasting was conducted per these recommendations. Most studies have indicated that this significantly increases the bond strength between the tooth structure and the composite material [40]. De Angelis et al. reported lower micro-tensile bond strength values for a composite material with dentin (6.46 MPa) compared with our values (18.65 MPa and 12.08 MPa) when treated with Al_2_O_3_ without silane application [37]. Other authors also obtained lower values as they did not use silane [43]. They used the same cement as us (iCem (Heraeus Kulzer)). Fuentes et al. did not observe a significant change in adhesive bond strength after silane application [42].

Thermal cycling is one of the most used methods that imitates clinical conditions and induces mechanical stress. Most studies in the literature did not evaluate the adhesive bond strength after thermal fatigue, limiting the data obtained [18,32,44]. In our study, the adhesive bond strength systematically decreased after thermal cycling, and the null hypothesis was rejected. This dependence was statistically significant in three studied groups: Signum ceramis/enamel, Cerasmart/enamel, and Cerasmart/dentin. In the Signum ceramis/dentin samples, a decrease in the µTBS was observed, but it was not statistically significant. The other authors also reported a substantial change in the adhesive bond strength after thermal cycling [40,49]. Three possible mechanisms by which temperature changes lead to a decrease in adhesive bond strength are detailed as follows: significant differences in the thermal expansion of different substrates (enamel/dentin/composite material) lead to the destruction of the adhesive bond [40]; the degradation of the cement due to the destruction of the filler/matrix bond; and the release of monomers from the cement upon a temperature increase [40]. Other authors claim that thermal cycling does not affect the adhesive bond strength [50]. Abo-Hamar et al. reported a significant change in enamel samples’ thermal cycling but not dentin samples [51]. The adhesive bond strength values we obtained show that it significantly decreased after temperature changes, except for in the composite material 1 group (Signum ceramis) cemented to dentin.

## 5. Conclusions

Within the limitations of this in vitro study, the conclusions drawn are as follows:

1. The thermal fatigue of the material led to decreased micro-tensile bond strength values for all the materials tested.

2. The Signum ceramis material bonded to dentin without thermocycling showed the best results in terms of bond strength.

3. The Cerasmart material bonded to dentin with thermocycling obtained the lowest values.

From a clinical point of view, it is preferred to save the enamel during preparation for indirect restorations due to the higher bond strength values.

## Figures and Tables

**Table 1:** Materials used in this study.

Material	Composition	Manufacturer
Signum ceramis	Monomer: 27 wt% multifunctional methacrylic acid esters, photo initiators, stabilizing agents, and inorganic pigments Filler: 73 wt% silicon dioxide and inorganic fillers	Heraeus Kulzer, Hanau, Germany
Cerasmart	Monomer: 29 wt% Bis-MEPP, UDMA, and DMA Filler: 71 wt% silica (20 nm) and barium glass (300 nm)	GC Europe, Leuven, Belgium
iCEM	Monomer: 51 wt% Di-, tri-, and multifunctional acrylates, initiators, and stabilizers Filler: 49 wt% filler	Heraeus Kulzer, Hanau, Germany
Silan IT	Ethanol (96%) and silane coupling agent (4%)	ITENA, Paris, France

Bis-MEPP, 2,2-bis(4-methacryloxypolyethoxyphenyl) propane; UDMA, urethane dimethacrylate; DMA, dimethacrylate.

**Table 2:** Statistical analysis results of the difference between micro-tensile strength in thermocycled and non-thermocycled specimens.

Group	Non-Thermocycled Mean ± SD (MPa)	Thermocycled Mean ± SD (MPa)	Group	Non-Thermocycled Mean ± SD (MPa)	Thermocycled Mean ± SD (MPa)
C_1_/E	12.22 ± 2.12 ^A, a^	8.53 ± 1.93 ^B, a^	C_1_/D	18.65 ± 3.98 ^A, a^	16.96 ± 3.66 ^A, a^
C_2_/E	14.58 ± 3.37 ^A, a^	10.60 ± 2.17 ^B, a^	C_2_/D	12.08 ± 2.53 ^A, b^	6.17 ± 1.28 ^B, b^

Legend: C_1_—Signum ceramis; C_2_—Cerasmart; E—enamel; and D—dentin. Groups with different letters indicate statistically significant differences. Different superscript uppercase letters in each row for each material indicate significant differences with aging (*p* < 0.05). Different superscript lowercase letters in each column for each composite material indicate significant differences (*p* < 0.05).

## Data Availability

The original contributions presented in the study are included in the article; further inquiries can be directed to the corresponding author.
